# Comparing Patients’ Opinions on the Hospital Discharge Process Collected With a Self-Reported Questionnaire Completed Via the Internet or Through a Telephone Survey: An Ancillary Study of the SENTIPAT Randomized Controlled Trial

**DOI:** 10.2196/jmir.4379

**Published:** 2015-06-24

**Authors:** Berengere Couturier, Fabrice Carrat, Gilles Hejblum, SENTIPAT Study Group

**Affiliations:** ^1^ Assistance Publique - Hôpitaux de Paris Unité de Santé Publique Hôpital Saint Antoine Paris France; ^2^ Sorbonne Universités, UPMC Univ Paris 06 UMR-S 1136 Institut Pierre Louis d'Epidémiologie et de Santé Publique Paris France; ^3^ INSERM U1136 Institut Pierre Louis d'Epidémiologie et de Santé Publique Paris France

**Keywords:** hospital information systems, patient-centered care, patient discharge, patient satisfaction, quality of health care

## Abstract

**Background:**

Hospital discharge, a critical stage in the hospital-to-home transition of patient care, is a complex process with potential dysfunctions having an impact on patients’ health on their return home. No study has yet reported the feasibility and usefulness of an information system that would directly collect and transmit, via the Internet, volunteer patients’ opinions on their satisfaction concerning the organization of hospital discharge.

**Objective:**

Our primary objective was to compare patients’ opinions on the discharge process collected with 2 different methods: self-questionnaire completed on a dedicated website versus a telephone interview. The secondary goal was to estimate patient satisfaction.

**Methods:**

We created a questionnaire to examine hospital discharge according to 3 dimensions: discharge logistics organization, preplanned posthospital continuity-of-care organization, and patients’ impressions at the time of discharge. A satisfaction score (between 0 and 1) for each of those dimensions and an associated total score were calculated. Taking advantage of the randomized SENTIPAT trial that questioned patients recruited at hospital discharge about the evolution of their health after returning home and randomly assigned them to complete a self-questionnaire directly online or during a telephone interview, we conducted an ancillary study comparing satisfaction with the organization of hospital discharge for these 2 patient groups. The questionnaire was proposed to 1141 patients included in the trial who were hospitalized for ≥2 days, among whom 867 eligible patients had access to the Internet at home and were randomized to the Internet or telephone group.

**Results:**

Of the 1141 patients included, 755 (66.17%) completed the questionnaire. The response rates for the Internet (39.1%, 168/430) and telephone groups (87.2%, 381/437) differed significantly (*P*<.001), but their total satisfaction scores did not (*P*=.08) nor did the satisfaction subscores (*P*=.58 for discharge logistics organization, *P*=.12 for preplanned posthospital continuity-of-care organization, and *P*=.35 for patients’ impressions at the time of discharge). The total satisfaction score (median 0.83, IQR 0.72-0.92) indicated the patients’ high satisfaction.

**Conclusions:**

The direct transmission of personal health data via the Internet requires patients’ active participation and those planning surveys in the domain explored in this study should anticipate a lower response rate than that issued from a similar survey conducted by telephone interviews. Nevertheless, collecting patients’ opinions on their hospital discharge via the Internet proved operational; study results indicate that conducting such surveys via the Internet yields similar estimates to those obtained via a telephone survey. The results support the establishment of a permanent dedicated website that could also be used to obtain users’ opinions on other aspects of their hospital stay and follow-up.

**Trial Registration:**

Clinicaltrials.gov NCT01769261; http://clinicaltrials.gov/ct2/show/NCT01769261 (Archived by WebCite at http://www.webcitation.org/6ZDF5bdQb).

## Introduction

Hospital discharge constitutes a pivotal step in a hospitalized patient’s management between the hospital and the return home. Discharge organization conditions the subsequent continuity of care, at least in part. Hospital discharge is a complex process that requires the participation of different actors (including the patient and his/her entourage) and the use of some documents and tools (eg, checklist and discharge package/brochure, patient records, discharge summaries, nursing discharge notes, medical prescriptions). The complexity of the process can explain the occurrence of organizational dysfunctions during its course that could potentially affect the health of individuals far after the hospitalization [1].

The opinion of health professionals on how discharge is organized has been the topic of several studies [2-6] and some of those studies also obtained patients’ opinions [3-6]. Hence, the patient has become a major player and a key partner at the center of the health care system [7]. Collecting his/her opinions on the organization of care seemed to be a relevant way to evaluate the quality of the process, for example, to ascertain its perceived quality as experienced by the patient [8,9]. Some studies evaluated the quality of the hospital discharge process based on patients’ opinions [10-15]. These studies involved various specific tools, administered at different times in regards to the hospital discharge moment, and based on various modes of administration: telephone, face-to-face interview, or self-administered paper questionnaires. None of those investigations sought the participation of patients via the Internet, even though its use by patients has increased markedly [16].

In this context, we undertook a study aimed at estimating the contribution of a system based on the direct transmission via a dedicated website of volunteer patients’ self-reported experiences on their own hospital discharge process. In particular, we wanted to explore the feasibility of such an information collection method by examining patients’ response rates and determining whether the information collected via the Internet was of similar quality as that obtained during a telephone interview, which is more difficult to conduct and more expensive to put in place.

First, we created a questionnaire concerning the hospital discharge process according to 3 dimensions: discharge logistics organization, preplanned posthospital continuity-of-care organization, and the patients’ impressions during discharge. Then, we collected patients’ responses to the questionnaire according to 2 different methods requiring the patient’s more- or less-active participation in reporting his/her opinions: patient’s direct transmission of information on a dedicated website or a classical telephone interview. We took advantage of the multicenter, randomized SENTIPAT trial (described in Methods) that had already randomized patients at discharge with Internet access at home to transmit personal information via the Internet or by telephone and a third group without Internet at home who were included to determine the representativeness of the randomized sample. For our ancillary study focused on the organization of hospital discharge, the primary objective was to compare the satisfaction of internet and telephone groups, hypothesizing no significant difference according to the data-collection method. The secondary goal was to analyze patients’ opinions on the different components of discharge process.

## Methods

### Overview

This investigation was conceived as an ancillary study of the multicenter, randomized SENTIPAT trial [17]. We took advantage of the trial to examine patients’ opinions on the organization of their hospital discharge. SENTIPAT participants were also asked to describe their experience with this process.

### General Description of the SENTIPAT Trial

This multicenter (5 adult acute care units in a Parisian teaching hospital participated voluntarily: digestive and general surgery, gastroenterology, hepatology, infectious diseases, and internal medicine), randomized trial focused on the evolution of patients’ health on returning home posthospitalization (follow-up duration: 6 weeks). The principal objective was to determine whether the information directly transmitted by the volunteer patients via a dedicated website was comparable with that obtained during a telephone interview. It was a noninferiority trial (the main judgment criterion was the percentage of patients reporting at least one clinically significant adverse event occurring during the 42 days after hospital discharge).

Consecutive patients with Internet access at home were eligible for inclusion. They were enrolled the day of hospital discharge and randomized into 2 groups (stratified by department): Internet or telephone follow-up. Patients not eligible (ie, with the same characteristics as those randomized but without Internet access at home) were also included at a ratio of 1:4 of noneligible to eligible patients.

Lastly, 2550 patients (510 from each unit) were initially planned. Between February 25, 2013 and September 8, 2014, we enrolled 2090 patients who were not cognitively impaired and did not have a behavioral disorder, who spoke and wrote French, and were returning home after an acute care hospitalization, regardless of the type of stay—standard hospitalization (scheduled or not) on weekdays only (maximum Monday to Friday or any combination thereof) or outpatient hospitalization (1 day)—and not opposed to participating in the trial.

### Characteristics of the Ancillary Study Focusing on the Discharge Process

#### Patients

This study concerned the 1141 patients included whose hospitalization lasted 2 or more days. The results of patients whose hospitalization lasted only 1 day (n=949) are reported in Multimedia Appendix 1; the organization of the discharge process after these very short stays was logically analyzed independently of those of longer duration.

#### Questionnaire Structure

Several tools have been developed to collect patients’ opinions on their hospital-to-home transition, including The Care Transition Measure [11], The Patient Continuity of Care Questionnaire [13], the Brief PREPARED instrument [12], or the Readiness for Hospital Discharge Scale [15]. None of these explore the 3 hospital discharge-related dimensions of interest to us. Therefore, we constructed a specific questionnaire, based on French national recommendations [18-20], and an international literature review (BC, FC, and GH, unpublished data, 2015).

The questionnaire explored 3 hospital discharge dimensions (henceforth referred to as 3 items) addressed in 17 questions (Table 1): discharge logistics organization (Q1, Q2, Q3, Q4, Q5, and Q11C-E), henceforth referred to as item 1; preplanned posthospital continuity-of-care organization (Q6, Q7, Q8, Q9, and Q10), henceforth referred to as item 2; and the patients’ impressions during discharge (Q11A, B, F, and G), henceforth referred to as item 3. Several questions (Q1, Q6 and Q8) specifically attempt to document specific aspects of the hospital-discharge process; the corresponding responses and a general discussion of the questionnaire are given in Multimedia Appendix 2.

#### Questionnaire Administration

For the telephone and noneligible patients, the hospital discharge questionnaire was administrated during a telephone interview with a clinical research technician 7 days after discharge (the appointment was scheduled the day of discharge), with a maximum of 3 attempts to contact them. For the Internet group, the same questionnaire was available on the dedicated website on the day of discharge (D0) and was completed directly online by the patient, who had been given oral and written instructions (information sheet) to connect for the first time 7 days postdischarge. “Reminders” were sent once weekly for 6 weeks after discharge to potential responders (of the Internet group) who had not completed the discharge questionnaire yet.

### Statistical Analyses

A patient satisfaction score concerning the hospital discharge process was calculated for each of the 3 items (see scores accorded in Table 1). Each subscore was calculated with a normalized sum of all the item questions (range 0-1), each question within a given item having the same weight, and the total score was computed (mean of the 3 subscores, range 0-1). Questions not concerning the patient (eg, Q11D did not concern patients who used public transportation) were not included in the calculations.

The global distribution of the responses for each discharge questionnaire question was assessed. Between-group qualitative variable (eg, sex) differences were compared with Fisher’s exact test and quantitative variables (eg, scores, age, hospitalization duration, and level of education that was considered as a 4-level ordinal variable) were compared with either the Mann-Whitney-Wilcoxon test or, for matched-paired data, Wilcoxon’s signed rank test. A *P* value ≤.05 defined significance of usual comparisons. However, the significance threshold was lower when a Bonferroni correction was applied for multiple comparisons, as indicated where appropriate. Missing data were taken into account as follows: nonresponding patients and incomplete questionnaires were excluded from the analyses. Also, for questions not concerning the patient, the response “nonapplicable” was used and they were not included in the analyses. All statistical computations used the R program.

**Table 1:** Discharge questionnaire and satisfaction scoring.

Item and question			Response choices	Score
**Discharge logistics organization**				
	Q1. Who decided when you would be discharged from the hospital?^a^		Staff physician of the unit in which you were hospitalized	
			Yourself	
			Your entourage	
			Your primary care physician	
			Other	
	Q2. Were you informed by a doctor or nurse of the modalities of your discharge (date, time, transportation: taxi, ambulance...)?		Yes	1
			No	0
	Q3. Were you consulted for the choice of discharge date and time?		Yes	1
			No	0
	Q4. Were the discharge date and time compatible with your return home and/or your entourage?		Yes	1
			No	0
	Q5. Was the scheduled discharge time respected?		Yes	1
			No	0
	Q11C. At discharge, what did you think of the time needed to obtain your medical and administrative documents?		Reasonable	1
			Too long	0
	Q11D. At discharge, what did you think of the time needed for your transportation to arrive?		Reasonable	1
			Too long	0
			Not concerned	NA
	Q11E. At discharge, did you have any difficulties dealing with the administrative discharge formalities?		Yes	0
			No	1
			Not concerned	NA
**Preplanned posthospital continuity-of-care organization**				
		Q6. At discharge, what documents were you given concerning your subsequent care? Check all that apply^a^	Prescription(s)	
			Discharge summary	
			Letter for primary care physician	
			Nursing discharge notes	
			Information booklet(s)	
			Appointment for a next hospitalization	
			Appointment for your next consultation	
			Appointment for your complementary test(s)	
			Other, specify	
			None	
		Q7. What did you think about the information provided by the medical or nursing staff when you received those discharge documents?	Highly satisfied	1
			Satisfied	0.75
			Poorly satisfied	0.25
			Not at all satisfied	0
			No information given	0
			Not concerned	NA
		Q8. Did you meet with a social worker during your hospitalization to discuss the organization of your return home?^a^	Yes	
			No	
		Q9. Was your primary care physician informed of your hospitalization?	Yes	1
			No	0
			I don’t know	0
			I don’t have a primary care physician	NA
		Q10. Did you have the phone number of the unit in which you were hospitalized (should you need it)?	Yes	1
			No	0
**Patient’s impressions of the hospital discharge process**				
		Q11A. At discharge, what did you think about its organization?	Well planned	1
			A sense of haste, upheaval	0
		Q11B. At discharge, what did you think about returning home?	Relieved	1
			Anxious	0
		Q11F. At discharge, what did you think about the information provided?	Sufficient	1
			Insufficient	0
		Q11G. At discharge, what did you think about the health care team’s availability and listening to you?	Sufficient	1
			Insufficient	0

^a^ Question intended to document the situation but not to be a score component.

## Results

### Patient Characteristics and Response Rates

**Figure 1:**
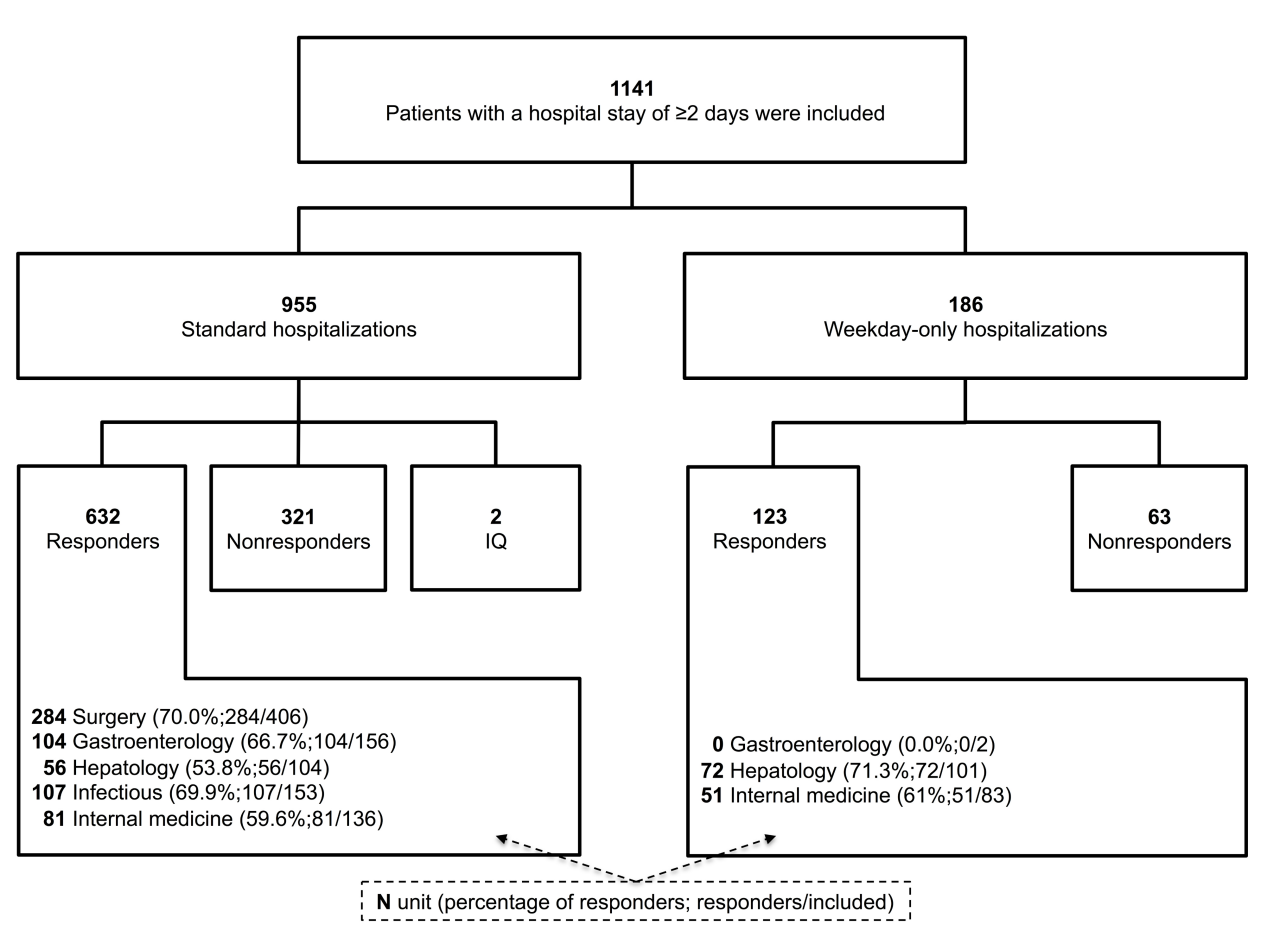
Flowchart of patients with a hospital stay of ≥2 days included in the SENTIPAT trial who responded or not to the discharge questionnaire according to the type of hospitalization and the recruitment unit. IQ: incomplete questionnaires; surgery: general and digestive surgery; infectious: infectious and tropical diseases.

A total of 755 (66.17%) completed discharge questionnaires were collected from the 1141 patients included after a hospital stay of 2 or more days (Figure 1). The relative contribution of each unit ranged from 13.41% (153/1141) to 35.58% (406/1141) and their response rates did not significantly differ from one unit to another (*P*=.08) and ranged from 60.3% (132/219) to 70.0% (284/406). Patients’ median age was 55 (IQR 39-66) years and 591 of 1141 (51.80%) were women. Hospitalization lasted a median 6 (IQR 3-10) days (median 7, IQR 4-11 and median 3, IQR 2-3 days for standard and weekday-only hospitalizations, respectively) (Table 2). Responders were significantly older than nonresponders (*P*<.001) for comparable sex distributions, level of education, and hospitalization durations. Internet, telephone, and noneligible group patients completed the questionnaire within median 6 (IQR 3-16), median 7 (IQR 7-9), and median 7 (IQR 7-8) days postdischarge, respectively, with respective Internet and telephone response rates of 39.1% (168/430) and 87.2% (381/437, *P*<.001). Noneligible patients were significantly older than telephone patients were (*P*<.001) and their response rate was significantly lower (75.2%, 206/274 vs 87.2%, 381/437, *P*<.001).

**Table 2:** Characteristics of patients with a hospital stay of 2 or more days.

Characteristic			Total	Responders	Nonresponders	*P*
**Group, n (%)**						
	All		1141	755 (66.17)	386 (33.83)	
	Internet		430	168 (39.1)	262 (60.9)	<.001^a^
	Telephone		437	381 (87.2)	56 (12.8)	
	Noneligible		274	206 (75.2)	68 (24.8)	<.001^b^
**Sex (male/female), n**						
	All		550/591	365/390	185/201	.90
	Internet		204/226	79/89	125/137	
	Telephone		228/209	198/183	30/26	
	Noneligible		118/156	88/118	30/38	
**Age (years), median (IQR)**						
	All		55 (39-66)	56 (41-67)	51 (34-64)	<.001
	Internet		51 (36-63)	55 (38-63)	48 (34-62)	
	Telephone		52 (34-64)	52 (36-64)	47 (30-66)	
	Noneligible		65 (52-74)	66 (55-75)	62 (49-72)	
**Length of hospital stay (days), median (IQR)**						
	All		6 (3-10)	6 (3-10)	6 (3-9)	.92
	Internet		6 (3-9)	5.5 (3-9)	6 (3-9)	
	Telephone		6 (3-10)	6 (3-10)	8 (3-10)	
	Noneligible		7 (3-11)	7 (3-12)	7 (3-9)	
**Level of education,** ^c^ **n (%)**						
	**All**					.95
		Level 1	223 (19.54)	152 (20.1)	71 (18.4)	
		Level 2	365 (31.99)	233 (30.9)	132 (34.2)	
		Level 3	150 (13.15)	103 (13.6)	47 (12.2)	
		Level 4	401 (35.14)	267 (35.4)	134 (34.7)	
		Do not wish to answer	2 (0.18)	0 (0.0)	2 (0.5)	
	**Internet**					
		Level 1	48 (11.2)	17 (10.1)	31 (11.8)	
		Level 2	138 (32.1)	51 (30.4)	87 (33.2)	
		Level 3	61 (14.2)	25 (14.9)	36 (13.7)	
		Level 4	182 (42.3)	75 (44.6)	107 (40.8)	
		Do not wish to answer	1 (0.2)	0 (0.0)	1 (0.4)	
	**Telephone**					
		Level 1	47 (10.8)	38 (10.0)	9 (16)	
		Level 2	139 (31.8)	121 (31.8)	18 (32)	
		Level 3	68 (15.6)	62 (16.3)	6 (11)	
		Level 4	182 (41.6)	160 (42.0)	22 (39)	
		Do not wish to answer	1 (0.2)	0 (0.0)	1 (2)	
	**Noneligible**					
		Level 1	128 (46.7)	97 (47.1)	31 (46)	
		Level 2	88 (32.1)	61 (29.6)	27 (40)	
		Level 3	21 (7.7)	16 (7.8)	5 (7)	
		Level 4	37 (13.5)	32 (15.5)	5 (7)	
		Do not wish to answer	0 (0.0)	0 (0.0)	0 (0)	

^a^ Internet vs telephone.

^b^ Noneligible vs telephone.

^c^ The levels of education were coded as follows: level 1, at most junior high school; level 2, high school; level 3, college; level 4, bachelor’s degree or above.

### Comparisons of Internet and Telephone Groups’ Satisfaction Scores

**Figure 2:**
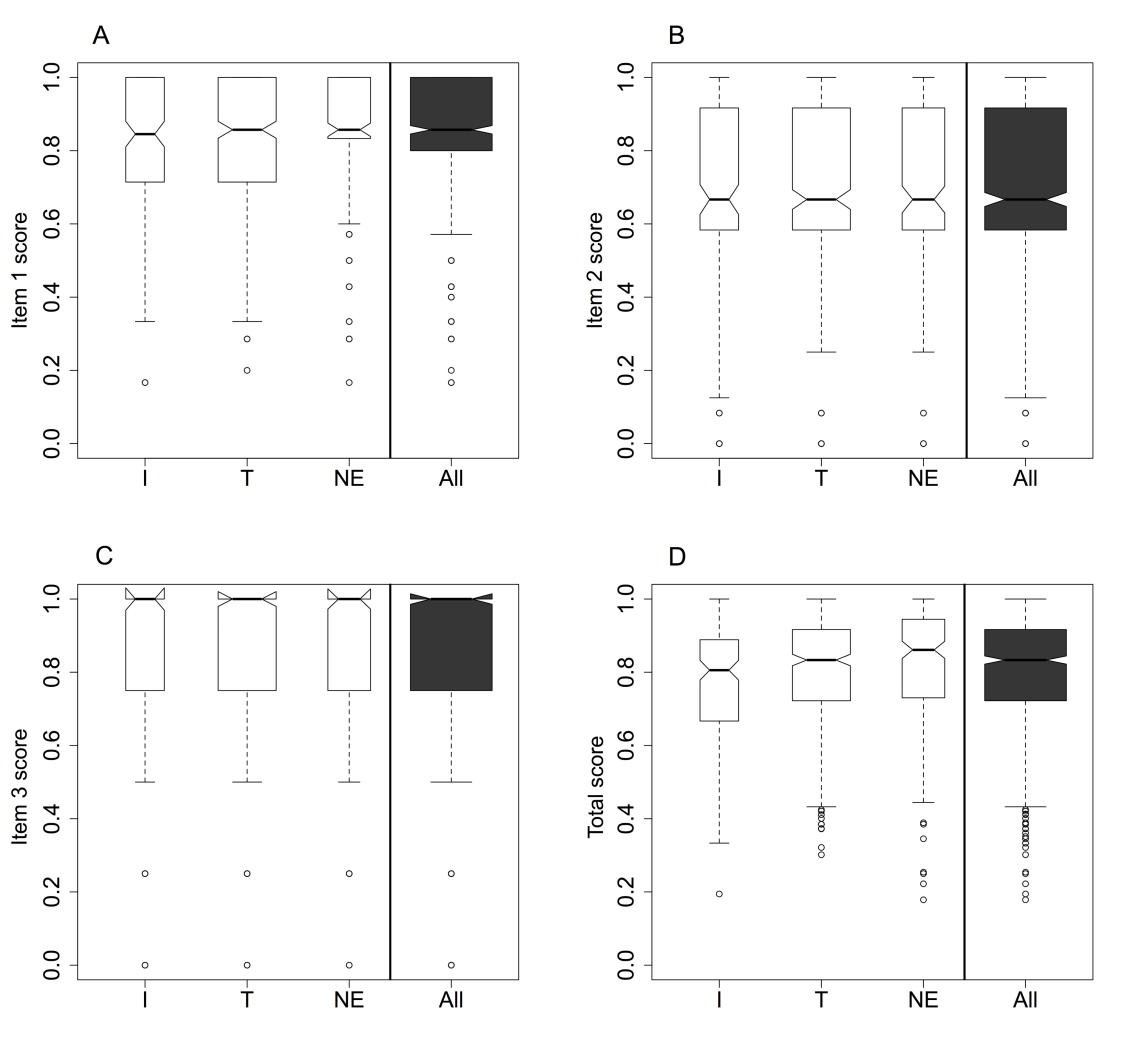
Box plots of score distributions according to Internet (I), telephone (T), or noneligible (NE) group. Item 1: discharge logistics organization; item 2: preplanned posthospital continuity-of-care organization; item 3: patient’s impressions of the hospital discharge process. The bold horizontal line is the median, the bottom and top borders of the boxes are 25th and 75th percentiles, respectively; the T-bar below and above the boxes represent 2.5th and 97.5th percentiles, respectively; the small white circles are outliers of the latter limits.

Box plot comparisons between the Internet and telephone groups for each of the 3 items or their total scores (Figure 2) revealed no important differences as confirmed by the corresponding statistical comparison results; all were associated with nonsignificant *P* values (Table 3). Moreover, the telephone and noneligible groups did not differ significantly for the total score or its 3 subscores.

**Table 3:** Distribution of satisfaction scores (percentiles) of the 755 responders with a hospital stay of 2 or more days according to group.

Score		Percentile							*P* ^a^
		5%	10%	25%	50%	75%	90%	95%	
**Total**									
	All	0.47	0.56	0.72	0.83	0.92	0.97	0.97	
	Internet	0.46	0.51	0.67	0.81	0.89	0.97	0.97	.08^b^
	Telephone	0.48	0.56	0.72	0.83	0.92	0.97	1	
	Noneligible	0.50	0.61	0.73	0.86	0.94	0.97	0.97	.03^c^
**Discharge logistics organization**									
	All	0.50	0.57	0.80	0.86	1	1	1	
	Internet	0.45	0.57	0.71	0.85	1	1	1	.58^b^
	Telephone	0.50	0.57	0.71	0.86	1	1	1	
	Noneligible	0.50	0.60	0.83	0.86	1	1	1	.06^c^
**Preplanned posthospital continuity-of-care organization**									
	All	0.33	0.33	0.58	0.67	0.92	1	1	
	Internet	0.25	0.33	0.58	0.67	0.92	1	1	.12^b^
	Telephone	0.33	0.33	0.58	0.67	0.92	1	1	
	Noneligible	0.33	0.33	0.58	0.67	0.92	1	1	.45^c^
**Patient’s impressions of the hospital discharge process**									
	All	0.25	0.50	0.75	1	1	1	1	
	Internet	0.25	0.43	0.75	1	1	1	1	.35^b^
	Telephone	0.25	0.50	0.75	1	1	1	1	
	Noneligible	0.50	0.50	0.75	1	1	1	1	.09^c^

^a^ Mann-Whitney-Wilcoxon tests with the corresponding Bonferroni correction for 2 comparisons: the telephone group was compared with the Internet and noneligible groups; all comparisons yielded nonsignificant *P* values.

^b^ Internet vs telephone.

^c^ Noneligible vs telephone.

### Satisfaction Scores for All Responders

#### Overview

The total satisfaction score was median 0.83 (IQR 0.72-0.92), with respective items 1-3 subscores of median 0.86 (IQR 0.8-1), median 0.67 (IQR 0.58-0.92), and median 1 (IQR 0.75-1). The box plots (Figure 2) for the entire population of responders differed in shape from one item to another. The Wilcoxon signed rank test confirmed that item 2 was significantly less well-rated than item 1 (*P*<.001), which was less well-rated than item 3 (*P*<.001) (Figure 2). The main characteristics associated with each of the 3 items are presented subsequently.

#### Item 1: Discharge Logistics Organization

Figure A3-1 in Multimedia Appendix 3 shows that 87.9% (664/755) of patients were informed of the modalities (eg, date and time, transportation) of their discharge; 53.1% (401/755) declared not having been consulted for the discharge date and time, and 92.6% (699/755) considered that those choices did not pose a problem. For 90.7% (685/755), the time was respected, 91.7% (676/737 excluding 18/755 patients not concerned) deemed the waiting time for medical and administrative discharge documents satisfactory, and 90.3% (650/720 excluding 35/755 patients not concerned) did not encounter difficulties completing administrative discharge procedures.

#### Item 2: Preplanned Posthospital Continuity-of-Care Organization

The explanations provided by the medical and/or nursing staff to accompany the document delivered at discharge (36/755 patients not concerned) were considered poorly satisfactory or totally unsatisfactory for 11.1% (80/719) and 16.0% (115/719) declared having received no explanations (Figure A3-2, Multimedia Appendix 3). According to 23.4% (177/755) of patients, their primary care physicians were not informed of their hospitalizations and 16.6% (125/755) did not know if they had been informed or not. In addition, 89.4% (675/755) had the telephone numbers of the unit in which they were hospitalized, if needed.

#### Item 3: Patients’ Impressions During the Discharge Process

Figure A3-3 (see Multimedia Appendix 3) describes patients’ impressions of the discharge process. Notably, 85.0% (642/755) thought that their discharge had been well planned. Those with the opposing opinion were significantly younger (*P*<.001). In addition, patients anxious about their return home (13.8%, 104/755) were significantly younger than those relieved at the idea of going home (*P*<.001). Moreover, 20.3% (153/755) of the patients thought they lacked information at discharge. Finally, 9.5% (72/755) of the patients judged hospital caregivers insufficiently available and that they listened insufficiently to the patient.

## Discussion

### Main Results and Comparison With Previous Findings

This study was designed to investigate patients’ opinions of the hospital discharge process with a questionnaire administered either by self-reporting directly online or by a traditional telephone interview. Response rates to our questionnaire showed that patients are willing to assume an active partnership role—87.2% for the telephone group (with Internet access at home) and 75.2% for noneligible patients (without Internet access at home)—values close to the average response rate for 13 studies that included a telephone inquiry to obtain patients’ impressions of hospital care delivered (ie, mean 70%, range 24%-91%) [21]. However, such interviews are more cumbersome and expensive to organize (eg, interviewer, scheduling of calls) and implement as routine practice over the long term. Responders were significantly older than nonresponders, with a similar observed pattern in the Internet and the telephone groups, and this might reflect individuals’ greater availability or interest in health care questions, which increases globally with age.

Given the rising availability and utilization of the Internet in all populations, this easy, low-cost approach as a means of collecting patients’ opinions might be an attractive alternative to telephone calls. In our study, the Internet patients’ response rate was lower (39.1%, 168/430) than the telephone patients’ response rate (87.2%, 381/437). In many respects, it is not surprising. For example, ignoring an invitation to actively enter personal data on a website is much easier than ignoring a live person who reaches another by phone. Nevertheless, the 39% response rate observed in our study exceeded that usually reached with Internet surveys, according to a meta-analysis of 39 studies (median 27%, range 14.5%-51%) [22]. Nonetheless, the information reported by online patients did not differ significantly from those collected by phone. Obtaining patients’ opinions on the discharge process is in-line with current initiatives to achieve a patient-centered health care system [23-25]. Our observations suggest that long-term implementation of an information system, similar to that developed for this study, would enable patients to directly transmit their hospital discharge experiences. The scores observed for the 3 dimensions of the discharge process explored herein indicate an overall positive patient perception: discharge logistics organization (median 0.86, IQR 0.8-1), preplanned posthospital continuity-of-care organization (median 0.67, IQR 0.58-0.92), and patients’ impressions of the process (median 1, IQR 0.75-1). Discharge logistics organization, in particular, was judged globally satisfactory even though approximately half of patients were not involved in the scheduling of their discharge date and time.

Pertinently, our results also identify several difficulties, notably hospital transmission of information to primary care physicians and the patient, and thereby also indicate how to potentially improve performance. Only 20.3% (153/755) of patients declared having left the hospital with a discharge summary and/or letter for their primary care physician. These observations agree with those previously reported by authors investigating discharge summary availability at the time of discharge for health care professionals responsible for posthospitalization continuity of care [26-33]. For example, in their review, Kripalani et al [28] indicated that only a median 14.5% (range 9%-20%) and median 52% (range 51%-77%) of primary care physicians had received discharge summaries 1 and 4 weeks after discharge, respectively.

In addition, more than a quarter of patients deemed the medical and/or nursing explanations of their discharge documents as poor or unsatisfactory, or had received none. Moreover, one-fifth of patients reported a lack of information at discharge. This absence of information and/or delivery of information not corresponding to patient expectations was also noted previously [13,27,34,35]. Other than the strict enumeration of the hospital discharge instructions provided to the patient by health professionals at discharge, the patient’s understanding of them is not always optimal [36,37], notably concerning medical treatments to be pursued [38-41], and can be underestimated by health professionals [42,43]. The findings of Horwitz et al [37] are particularly interesting because despite the demonstration of a gap between the information given to the patients and their understanding of it, the patients “were uniformly positive in their assessment of discharge care” as in our study. In a 2014 systematic revue [44] of 36 studies targeting patients’ opinions on quality of care, only 2 addressed the quality of the discharge process [45,46] and they reported globally positive impressions. However, 2 other studies [10,14] examined the association between patient satisfaction with the discharge process and the hospital readmission rate within 30 days, an important health outcome measure, and found it to be significant suggesting this impression reflects, at least in part, the quality of hospital care. Nevertheless, the associated performance differences were relatively modest, thereby suggesting that improving patient satisfaction with discharge organization would also have a minor impact on health in terms of solid outcome measures.

### Limitations

Our study has several limitations. Inherent to studies requiring an active participation, responders constitute an intrinsic biased selection sample of patients. An example of a demographic status significantly associated with the responder status is age. Moreover, differences are introduced by the questionnaire administration mode (Internet vs telephone). The response rate observed in the Internet group is somewhat disappointing, but another study on patient satisfaction also reported similar response rates: 34% and 78% in the Internet and telephone groups, respectively [47]. Nevertheless, even if a similar response rate had been observed in the telephone and Internet groups, this would not exclude different selection biases from one group to the other (eg, inherent to the mode of administration). The major result of the study is that despite the biases of this study (potential or not), the estimates issued from the 2 groups are very similar; therefore, an Internet-based survey in the domain investigated should be considered as a useful alternative to a “reference” telephone survey. Nonetheless, collecting patients’ opinions via the Internet, as done in this study, has numerous advantages. First, unlike the telephone interview that inserts a third person and a potential information bias (survey subjectivity), resorting to the Internet allows self-administration of the questionnaire, triggering the patient’s active participation. Finally, this method of data collection is less costly than managing a telephone cohort and yields comparable information. However, the similar scores in the telephone and Internet mode of administration observed in this study are based on a particular newly developed questionnaire deployed in a given patient population; therefore, this limits the generalizability (external validity) of the results.

### Conclusions

The results of this study advocate for establishing a permanent information system that would enable volunteering patients to express their opinions concerning hospital discharge. Such an information system could also be used for other management issues related to health care organization. Those planning to design similar surveys via the Internet should anticipate a response rate comparable to that observed in the present study. Nevertheless, the concept of sentinel patient explored in this study could constitute, in the future, an essential tool in a patient-centered approach to the organization of care.

## Appendix

Multimedia Appendix 1 Analysis of discharge-questionnaire responses of patients with 1-day hospital stays.

Multimedia Appendix 2 Additional information about the discharge questionnaire.

Multimedia Appendix 3 Other figures supplementing the main manuscript.

Multimedia Appendix 4 CONSORT-EHEALTH checklist V1.6.1 [48].
