# Supplementary material for: Comparing Patients’ Opinions on the Hospital Discharge Process Collected With a Self-Reported Questionnaire Completed Via the Internet or Through a Telephone Survey: An Ancillary Study of the SENTIPAT Randomized Controlled Trial
Source: J Med Internet Res. 2015 Jun 24;17(6):e158. doi: 10.2196/jmir.4379 (PMC4526961; doi:10.2196/jmir.4379)

MULTIMEDIA APPENDIX 1

Analysis of Discharge-Questionnaire Responses of Patients with 1-Day Hospital Stays

Patient Characteristics and Response Rates

Table A1-1 reports the response rates and characteristics of patients with 1-day hospital stays for all types of hospitalizations combined. Among 949 patients included, 631 (66.5%) returned completed questionnaires (Figure A1-1). The participation of the different units ranged from 5.5% (52/949) to 37.6% (357/949) and response rates ranged from 64.4% (67/104) to 68.1% (243/357) (*P*=.92). The median age of included patients was 45 (IQR 34-55) years and 590/949 (62.2%) were men. The ages, levels of education, and male/female ratios for responders and nonresponders were comparable. The response rate for Internet patients was significantly lower that of the telephone group (43.4% (178/410) versus 85.1% (343/403), respectively; *P*<.001) and the time to questionnaire completion online was median 6 (IQR 3-13) days after discharge (the corresponding times for the telephone and noneligible groups, respectively, were median 7 (IQR 7-9) and median 7 (IQR 7-8) days post-hospitalization). Noneligible patients were significantly older than telephone-group participants (*P*<.001) and the response rates for the 2 groups were comparable 80.9% (110/136) versus 85.1% (343/403), *P*=.28).

Table A1-1

Characteristics of the patients with a 1-day stay.

Characteristic	Total	Responders	Nonresponders	*P* value
Group, n (%)				
Total	949	631 (66.5)	318 (33.5)	
Internet	410	178 (43.4)	232 (56.6)	<.001[a]
Telephone	403	343 (85.1)	60 (14.9)	
Noneligible	136	110 (80.9)	26 (19.1)	.28[b]
Sex (Males/Females)				
Total	590/359	389/242	201/117	.67
Internet	258/152	112/66	146/86	
Telephone	255/148	217/126	38/22	
Noneligible	77/59	60/50	17/9	
Age (years), median (IQR)				
Total	45 (34-55)	45 (35-55)	44 (31-54)	.052
Internet	45 (33-54)	46 (35-54)	44 (31-54)	
Telephone	41 (32-52)	42 (33-53)	39 (30-48)	
Noneligible	54 (45-65)	53 (44-65)	57 (48-65)	

(Table A1-1 continued)

Characteristic	Total	Responders	Nonresponders	*P* value
Level of education[c], n (%)				
All				0.10
Level 1	118 (12.4)	79 (18.3)	39 (8.2)	
Level 2	274 (28.9)	173 (40.0)	101 (21.1)	
Level 3	141 (14.9)	55 (12.7)	47 (9.8)	
Level 4	412 (43.4)	122 (28.2)	290 (60.7)	
Do not wish to answer	3 (0.3)	3 (0.7)	0 (0.0)	
Missing data	1 (0.1)	0 (0.0)	1 (0.2)	
Internet				
Level 1	28 (6.8)	7 (3.9)	21 (9.1)	
Level 2	130 (31.7)	51 (28.7)	79 (34.1)	
Level 3	71 (17.3)	30 (16.9)	41 (17.7)	
Level 4	181 (44.1)	90 (50.6)	91 (39.2)	
Do not wish to answer	0 (0.0)	0 (0.0)	0 (0.0)	
Missing data	0 (0.0)	0 (0.0)	0 (0.0)	

(Table A1-1 continued)

Characteristic	Total	Responders	Nonresponders	*P* value
Level of education[c], n (%)				
Telephone				
Level 1	31 (7.7)	25 (7.3)	6 (10)	
Level 2	99 (24.6)	88 (25.7)	11 (18)	
Level 3	59 (14.7)	45 (13.1)	14 (23)	
Level 4	213 (52.9)	185 (53.9)	28 (47)	
Do not wish to answer	0 (0.0)	0 (0.0)	0 (0)	
Missing data	1 (0.2)	0 (0.0)	1 (2)	
Noneligible				
Level 1	59 (43.4)	47 (42.7)	12 (46)	
Level 2	45 (33.1)	34 (30.9)	11 (42)	
Level 3	11 (8.1)	11 (10.0)	0 (0)	
Level 4	18 (13.2)	15 (13.6)	3 (12)	
Do not wish to answer	3 (2.2)	3 (2.7)	0 (0)	
Missing data	0 (0.0)	0 (0.0)	0 (0)	

[a]Internet vs telephone

[b]Noneligible vs telephone

[c]The levels of education were coded as follows: Level 1, at most junior high school; Level 2, high school; Level 3, college; Level 4: bachelor’s degree or above.

Figure A1-1

Flowchart of patients included in the SENTIPAT trial with a 1-day hospital stay who responded or not to the discharge questionnaire according to the type of hospitalization and the recruitment unit. IQ: incomplete questionnaires; Surgery: general and digestive surgery; Infectious: Infectious and tropical diseases.

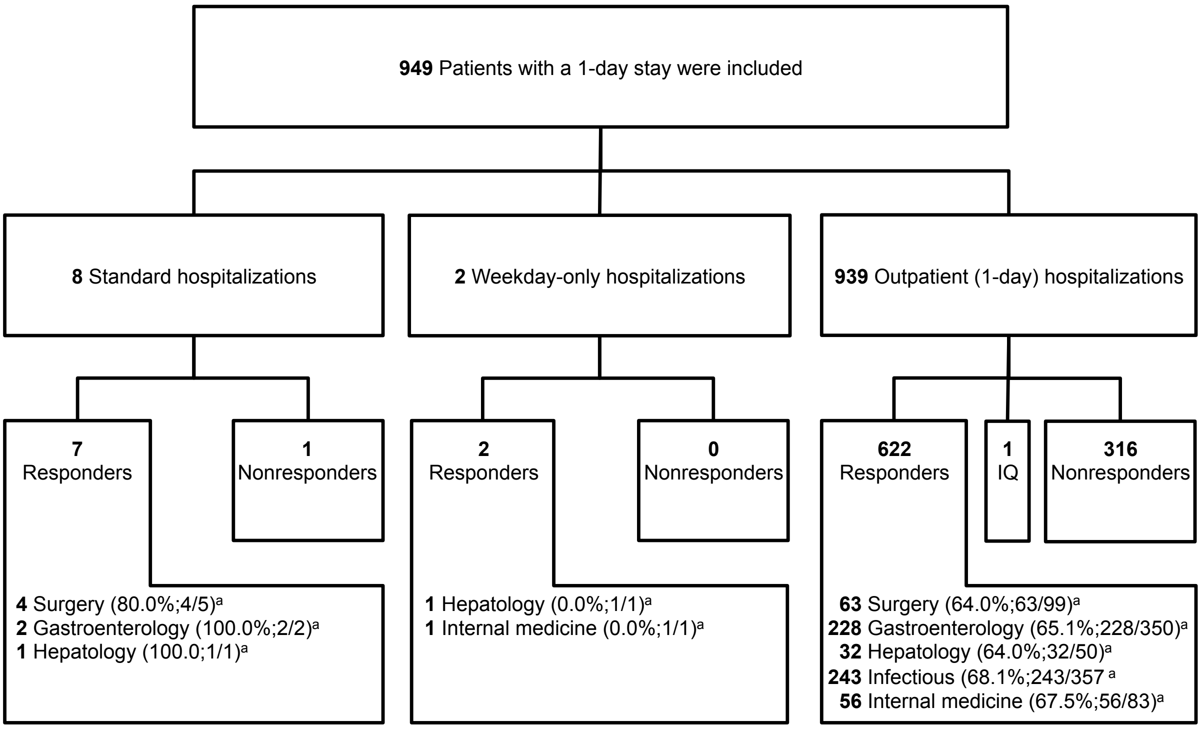

[a]**number of responders** unit (percentage of responders, responders/included)

Comparisons of the Different Satisfaction Scores Between the Telephone and Internet Groups

The graphic comparisons between the telephone- and Internet-group scores for the 3 items indicated no marked differences (Figure A1-2A, B and C), as confirmed by the corresponding statistical test results (Table A1-2). Notably, no significant difference was found between the total scores of these 2 groups (Figure A1-2D and Table A1-2).

Graphic differences between the telephone and noneligible groups were observed for the item-2 (Figure A1-2, B) and total scores (Figure A1-2, D) that were confirmed statistically (Table A1-2).

Figure A1-2

Box plots of score distributions according to All (all 631 responders), Internet (I), telephone (T) or noneligible (NE) group. Item 1: discharge logistics organization; item 2: preplanned posthospital continuity-of-care organization; item 3: patient's impressions of the hospital discharge process. The bold horizontal line is the median, the bottom and top borders of the boxes are 25th–75th percentiles; and the T-bar represents 2.5th–97.5th percentiles; the small white circles are outliers of the latter limits.

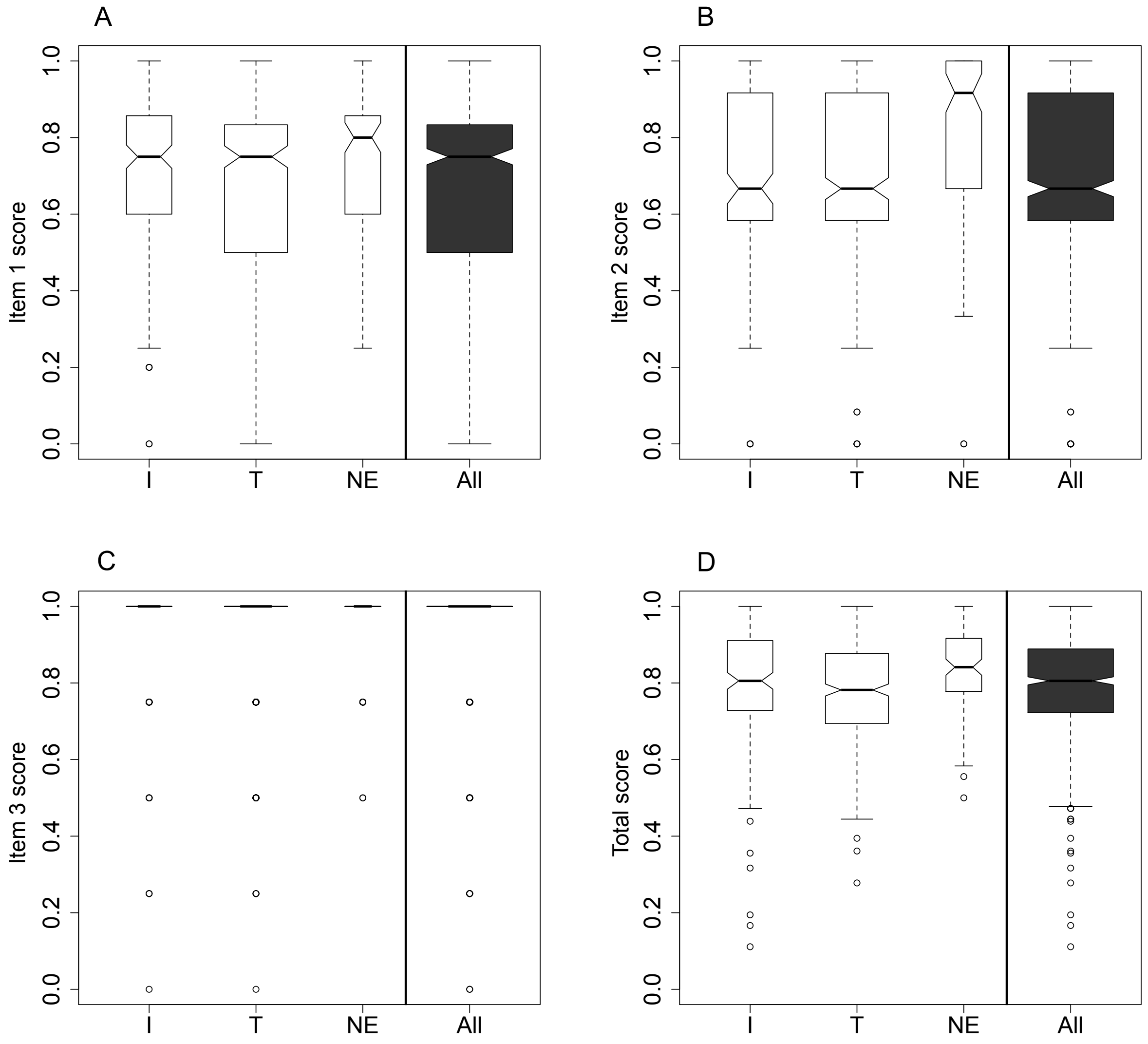

Table A1-2

Distribution of the satisfaction scores (percentiles) of the 631 responders with a 1-day stay according to group.

Score	Percentiles							*P* value[a]
Group	5%	10%	25%	50%	75%	90%	95%	
**Total**								
All	0.56	0.61	0.72	0.81	0.89	0.94	0.97	
Internet	0.53	0.61	0.73	0.81	0.91	0.96	1	.09[b]
Telephone	0.56	0.61	0.69	0.78	0.88	0.93	0.95	
Noneligible	0.61	0.68	0.78	0.84	0.91	0.94	0.97	<.001[c]
**Item 1**								
All	0.33	0.33	0.50	0.75	0.83	1	1	
Internet	0.25	0.33	0.60	0.75	0.85	1	1	.15[b]
Telephone	0.33	0.33	0.50	0.75	0.83	1	1	
Noneligible	0.33	0.33	0.60	0.80	0.86	1	1	.03[c]
**Item 2**								
All	0.33	0.42	0.58	0.67	0.92	1	1	
Internet	0.33	0.42	0.58	0.67	0.92	1	1	.81[b]
Telephone	0.33	0.33	0.58	0.67	0.92	1	1	
Noneligible	0.33	0.50	0.67	0.92	1	1	1	<.001[c]
**Item 3**								
All	0.75	0.75	1	1	1	1	1	
Internet	0.71	0.75	1	1	1	1	1	.53[b]
Telephone	0.53	0.75	1	1	1	1	1	
Noneligible	0.75	0.75	1	1	1	1	1	.11[c]

All, all 631 responders.

[a]Mann–Whitney–Wilcoxon tests with the corresponding Bonferroni correction for 2 comparisons: the telephone group was compared with the Internet and noneligible groups; only noneligible versus telephone *P* values were significant for total score and item 2.

[b]Internet vs telephone

[c]Noneligible vs telephone

Satisfaction Scores for All Responders

The total satisfaction score was median 0.81 (IQR 0.72-0.89). The item-1 discharge-logistics organization, item-2 preplanned post-hospitalization continuity-of-care organization and item-3 patients' impressions scores, respectively, were: median 0.75 (IQR 0.5-0.83), median 0.67 (IQR 0.58-0.92) and median 1 (IQR 1-1) (Table A1-2). The shapes of the box plots (Figure A1-2, black) from one item to another for the entire population differed. Wilcoxon signed-rank tests confirmed that item 2 (Figure A1-2B) was significantly less well graded (P<.001) than item 1 (Figure A1-2A), which was significantly less well graded than item 3 (Figure A1-2C) (P<.001).

The principal characteristics associated with each of the 3 items are addressed below.

Item 1: Discharge-Logistics Organization (Q2, Q3, Q4, Q5, Q11C–E)

As shown in Figure A1-3, 58.5% (369/631) of the patients were informed of the modalities (time, transportation...) of their discharge (Q2). It is of interest to note that, although 80.5% (508/631) of the patients stated not having been consulted for the choice of discharge time (Q3), 92.4% (583/631) felt that the time chosen (whether or not they had been consulted) did not pose a problem (Q4).

For 87.8% (352/401; excluding 230/631 not concerned) of the patients, the discharge time was respected (Q5). For 95.5% (448/469, 162/631 patients not concerned), the time waiting for medical and administrative discharge documents (Q11C) was satisfactory and 93.5% (100/107, 524/631 patients not concerned) considered the time needed for transportation to arrive (Q11D) was satisfactory. In addition, 96.2% (401/417, 214/631 patients not concerned) of the patients encountered no difficulties with the administrative discharge process (Q11E).

Figure A1-3

Item 1 score: discharge-logistics organization (Q2, Q3, Q4, Q5, Q11C–E).

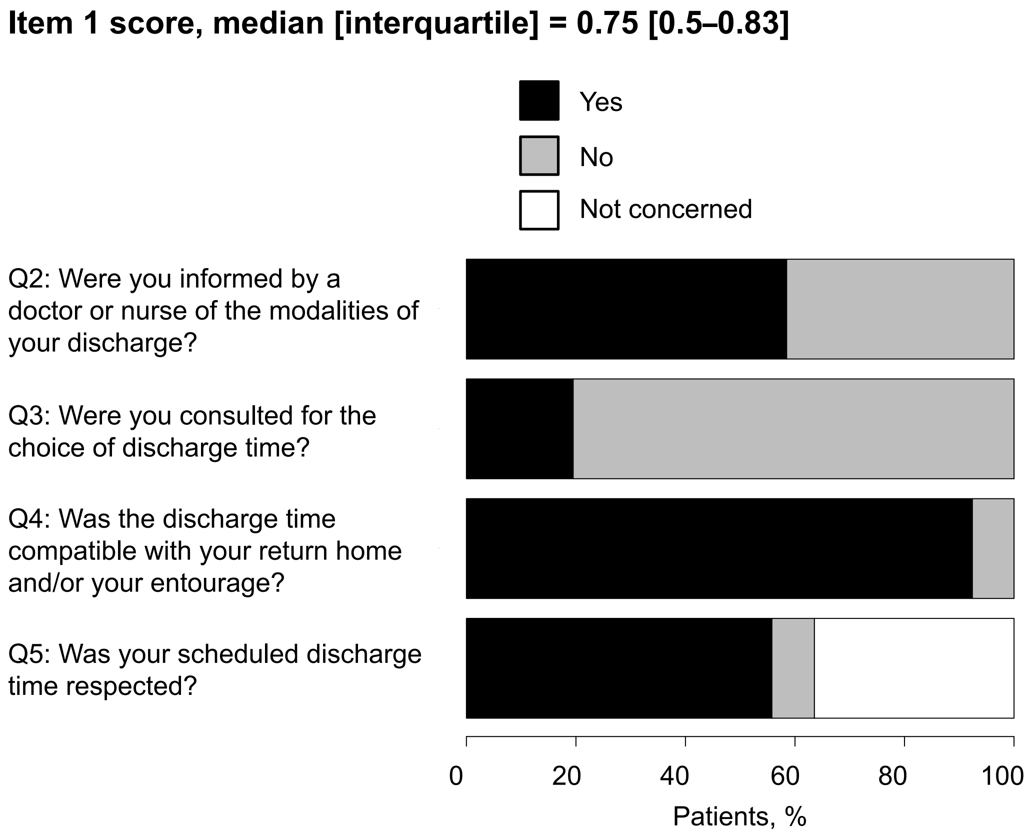

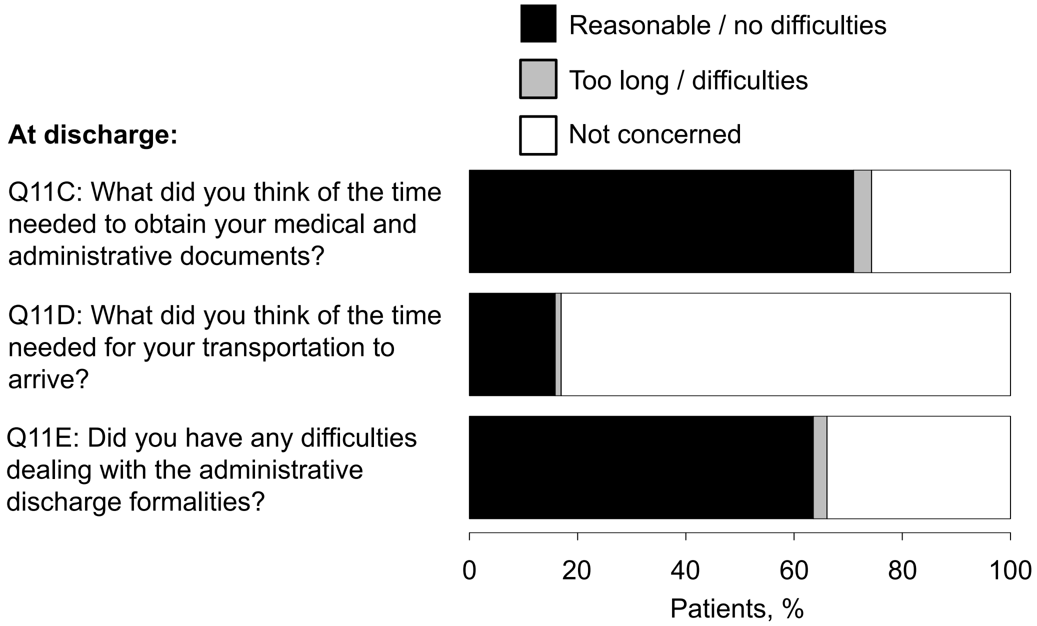

Item 2: Preplanned Post-Hospitalization Continuity-of-Care Organization (Q7, Q9, Q10)

Concerning the continuity of care after discharge (Figure A1-4), the information provided by the medical and/or nursing team with the discharge documents (Q7, 137/631 patients not concerned) was considered satisfactory or highly satisfactory by 76.5% (378/494) of the patients. However, it should be emphasized that 20.2% (100/494) declared having received no information, 28.2% (178/631) indicated that their primary-care physician was not informed of their hospitalization and 11.1% (70/631) did not know if their primary-care physician had been informed or not (Q9). Finally, 94.6% (597/631) of the patients had the telephone number of the unit in which they had been hospitalized, if needed (Q10).

Figure A1-4

Item 2 score: preplanned post-hospitalization continuity-of-care organization (Q7, Q9, Q10).

Item 2 score, median [interquartile] = 0.67 [0.58–0.92]

Q7: What did you think about the information provided by the medical or nursing staff when you received your discharge documents?

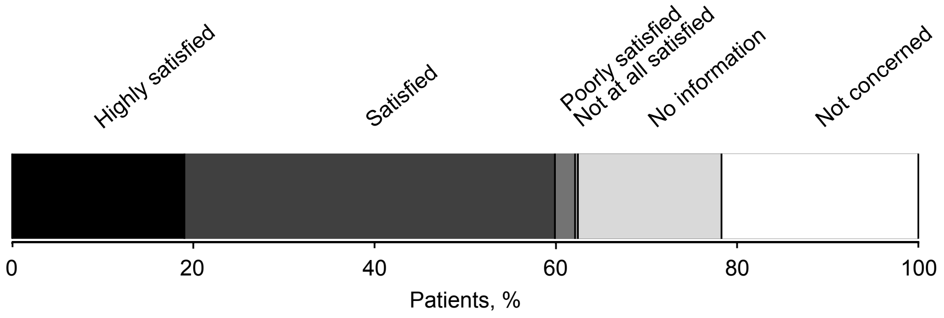

Q9: Was your primary-care physician informed of your hospitalization?

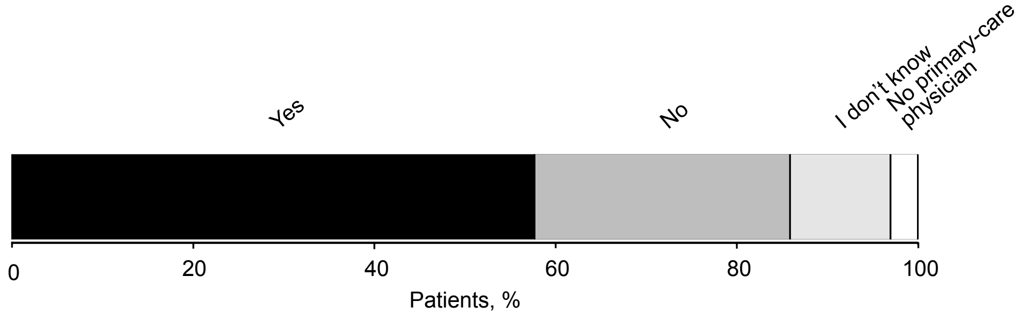

Q10: Did you have the phone number of the unit in which you were hospitalized?

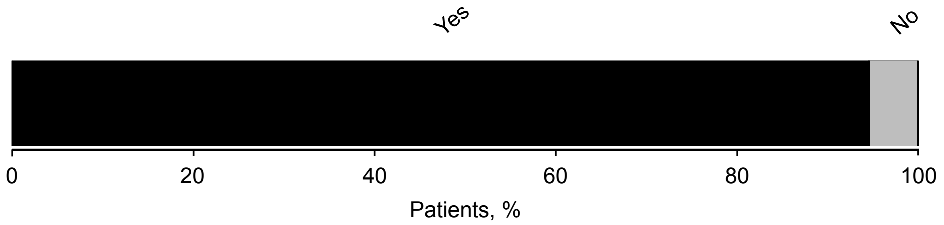

Item 3: Patients' Impressions of the Hospital-Discharge Process (Q11A, B, F, G)

Figure A1-5 illustrates the patients' impressions at the time of discharge. Indeed, 93.3% (589/631) judged their hospital discharge well-planned (Q11A), 96.0% (606/631) had a feeling of relief at the idea of going home (Q11B), 91.9% (580/631) thought the information provided by the healthcare staff was satisfactory (Q11F) and 97.3% (614/631) judged hospital caregivers were sufficiently available, and that they listened sufficiently to the patient (Q11G).

Figure A1-5

Item 3 score: patients' impressions of the hospital-discharge process (Q11A, B, F, G).

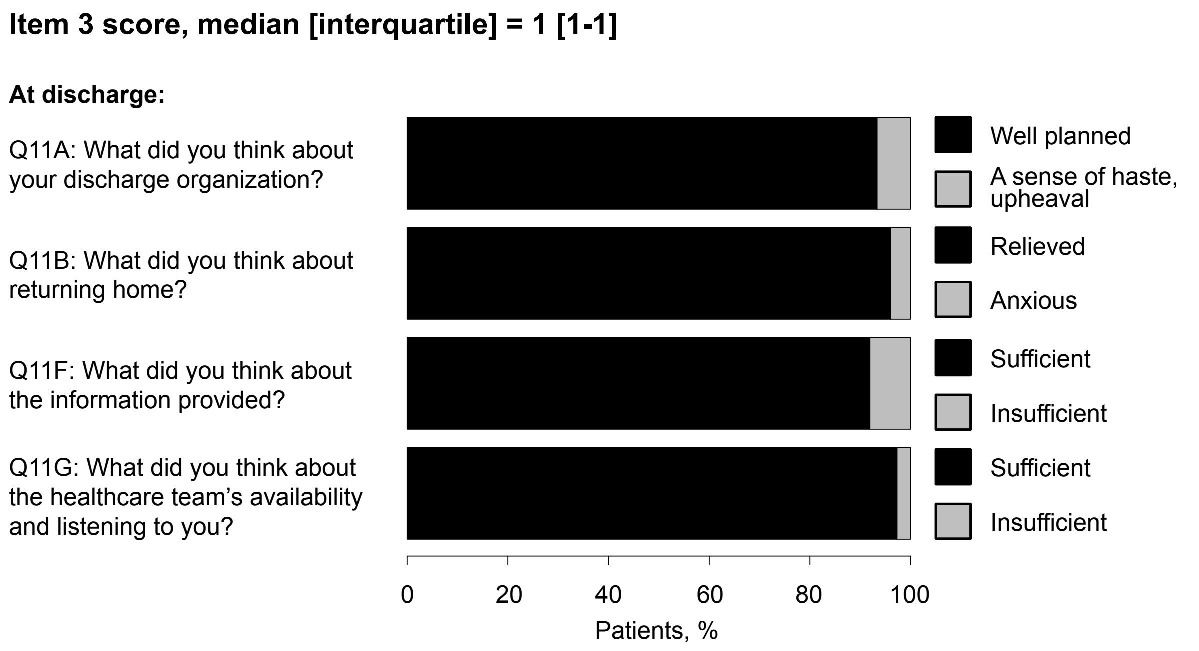

Comparisons Between Hospital Stays of 1 Versus ≥2 Days

Independent analyses were made taking into account the patient profiles and types of hospitalizations. However, some comment can be made concerning the results obtained.

Baseline Characteristics

Patients included in the study after 1-day hospitalization were significantly younger than those included after ≥2-days stays (median 45 (IQR 34-55) versus median 55 (IQR 39-66) years, respectively; $P<.001$).

Response Rate

The 2 populations had comparable response rates: 66.5% (631/949) for 1-day stays versus 66.17% (755/1141) for longer hospitalizations.

Satisfaction Scores

Comparisons of scores between groups

Although no significant between-group differences were observed for responders after a ≥2-day hospitalization, regardless of the score considered (item 1, 2 or 3 or total score), among responders after 1-day stays, noneligible patients were significantly more satisfied than the telephone group for item-2 and total scores (Table A1-2).

Score comparisons between patients with 1- or ≥2-days hospital stays (Figure A1-6)

Two major differences are worth mentioning for patients hospitalized for a single day: 1) they were less satisfied than the others about their discharge organization; and 2) their overall impression during discharge was very positive. However, it must be kept

in mind that the discharge questionnaire was mainly adapted to patients who had hospital stays ≥2 days long: as illustrated by item 1, substantial percentages of patients with 1-day stays [ranging from 25.7% (162/631, Q11C) to 83.0% (524/631, Q11D)] were not concerned for 4 out of 7 questions (Q5, Q11C–E).

Figure A1-6

Box plots of score distributions comparing 1-day (n=631) versus ≥2-days (n=755) hospital stays. Item 1: discharge logistics organization; item 2: preplanned posthospital continuity-of-care organization; item 3: patient’s impressions of the hospital discharge process.

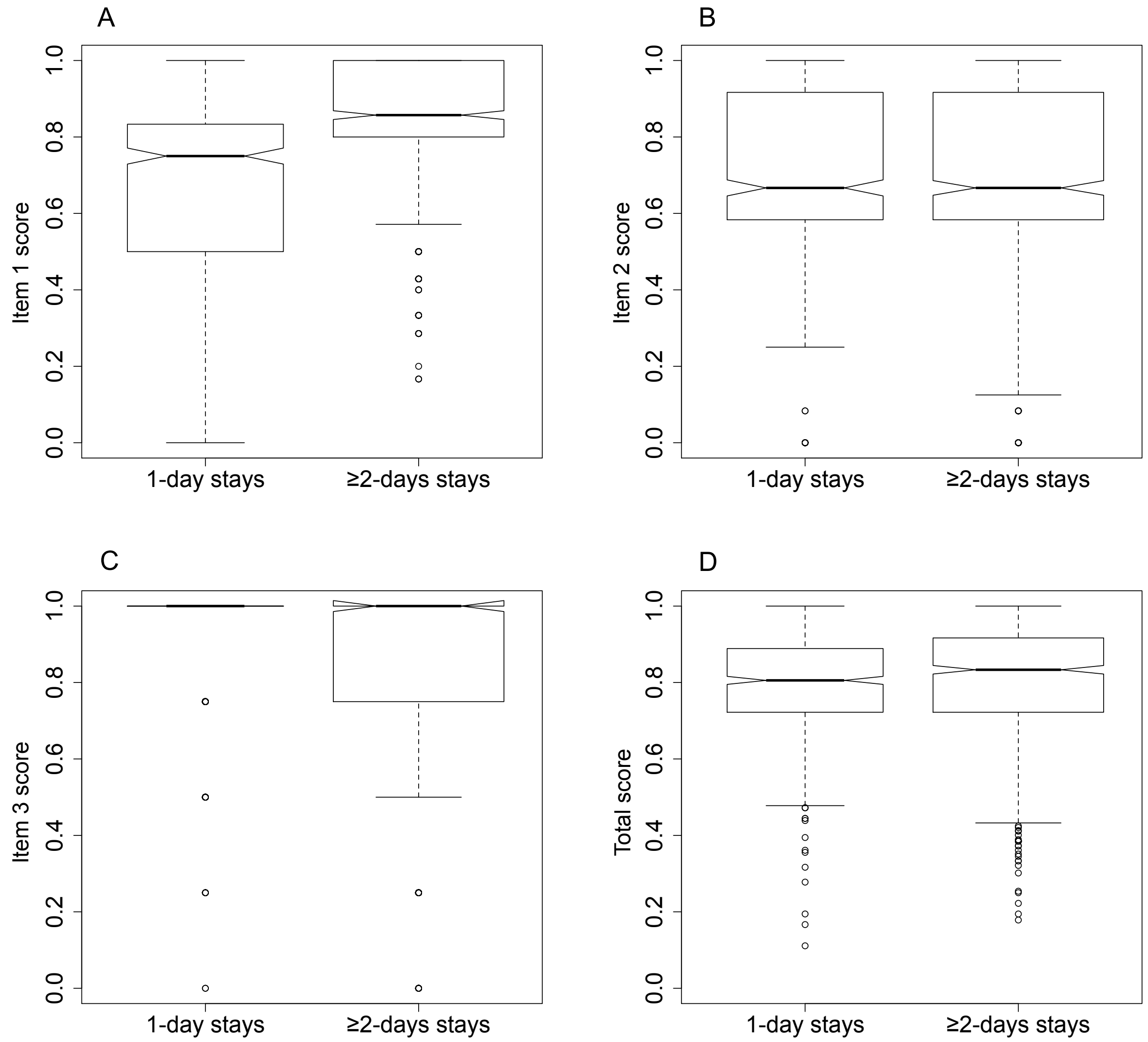

Supplement: Multimedia Appendix 1 [file jmir_v17i6e158_app1.pdf]
