# Supplementary material for: Comparing Patients’ Opinions on the Hospital Discharge Process Collected With a Self-Reported Questionnaire Completed Via the Internet or Through a Telephone Survey: An Ancillary Study of the SENTIPAT Randomized Controlled Trial
Source: J Med Internet Res. 2015 Jun 24;17(6):e158. doi: 10.2196/jmir.4379 (PMC4526961; doi:10.2196/jmir.4379)
Supplement: Multimedia Appendix 2 [file jmir_v17i6e158_app2.pdf]

MULTIMEDIA APPENDIX 2

Additional Information About the Discharge Questionnaire

Questions Not Included in the Calculation of the Total Satisfaction Score

The discharge questionnaire, specifically created for the study, included 3 questions that were not taken into consideration for the computation of the total satisfaction score because the questions were intended to document certain facets of the discharge process: Q1: who initiated the hospital discharge; Q6: inventory of the discharge documents given to the patient; and Q8: whether or not a social worker was involved in organizing the return home.

Stays ≥2 Days

For 684 (91.0%) of the 752 responding patients (3 not concerned), the hospital staff physician initiated the discharge and, for 43/752 (5.7%) patients, the patients or their entourage were responsible (Q1). At the time of discharge, 658/755 (87.2%) patients had a discharge prescription, 430/755 (57.0%) had a scheduled consultation and 153/755 (20.3%) had a discharge summary (76/755 (10.1%)) or letter to the primary-care physician (94/755 (12.5%)). Finally, 60/755 (7.9%) patients declared not have met with a social worker during their hospitalization to address the organization of their discharge (Q8).

1-Day Stays

Questions Q1, seeking the person initiating the hospital discharge did not concern the majority of hospitalized patients (613/631) for this type of stay. At the time of

discharge (Q6), 324/631 (51.3%) patients had a discharge prescription, 211/631 (33.4%) had a discharge summary, 206/631 (32.6%) had a scheduled consultation, 196/631 (31.1%) had a future hospitalization scheduled and 143/631 (22.7%) declared having been discharged without any document. Finally, Q8, which explored the input of social workers to organize discharge, is not adapted to 1-day stays; only 5/631 responders stated having met with a social worker.

Limitations and Possible Improvements of the Discharge Questionnaire

This first solid experience with the questionnaire led us to propose the following modifications to improve this tool for future use.

For Q1, "Who was your main contact at the time of your discharge?" would be more pertinent.

Q7 could be completed by a complementary question, "What discharge information did you need?", to identify the patient-perceived missing information (notably when patients stated not having received any).

For Q9, which raise a temporal issue, reformulations, for example, "When you were hospitalized, did you know if your primary-care physician was informed of your admission?", would be better.

Similarly, Q10 could be completed by the following complementary question: "If yes, were you able to contact the unit easily or with difficulty?". This formulation would enable the study of the real availability of telephone assistance, more than simply providing the telephone number.

Collecting patients' opinions on the content and structure of the discharge questionnaire might also enhance its value.

In the end, the current questionnaire was not always adapted to patients hospitalized for only 1 day. In particular, our study results showed that a very high percentage of them were not concerned by questions Q5 and Q11C–E. This advocates for adapting the questionnaire to such very short stays, especially in regards to the current rapid spreading of short-term outpatient services (eg, outpatient hospitalization, ambulatory surgery).
