# Supplementary material for: Comparing Patients’ Opinions on the Hospital Discharge Process Collected With a Self-Reported Questionnaire Completed Via the Internet or Through a Telephone Survey: An Ancillary Study of the SENTIPAT Randomized Controlled Trial
Source: J Med Internet Res. 2015 Jun 24;17(6):e158. doi: 10.2196/jmir.4379 (PMC4526961; doi:10.2196/jmir.4379)

MULTIMEDIA APPENDIX 3

Other figures supplementing the main manuscript.

Figure A3-1

Item 1 score: discharge-logistics organization (Q2, Q3, Q4, Q5, Q11C–E).

Item 1 score, median [interquartile]: 0.86 [0.8–1]

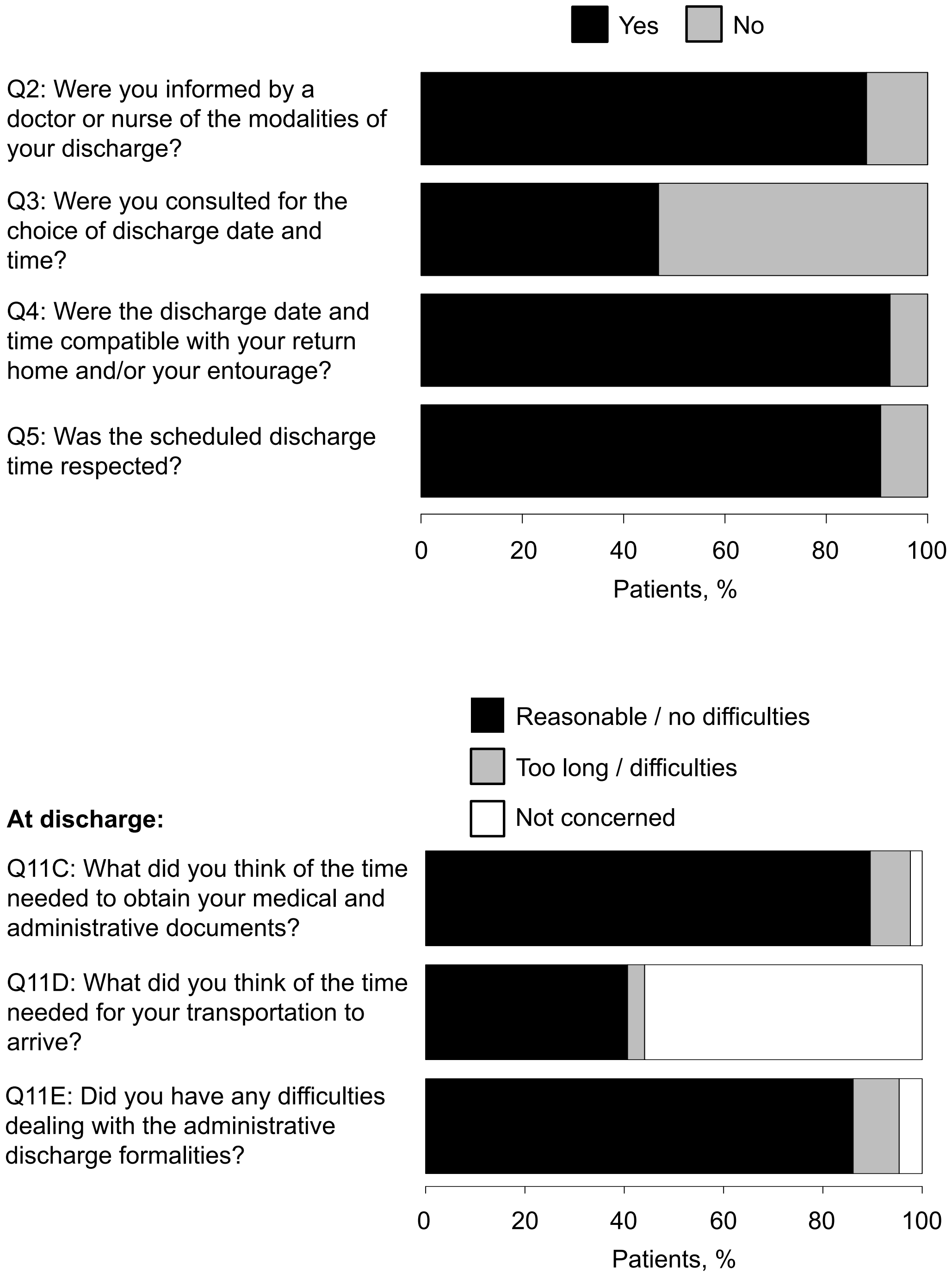

Figure A3-2

Item 2 score: preplanned post-hospitalization continuity-of-care organization (Q7, Q9, Q10).

Item 2 score, median [interquartile] = 0.67 [0.58–0.92]

Q7: What did you think about the information provided by the medical or nursing staff when you received your discharge documents?

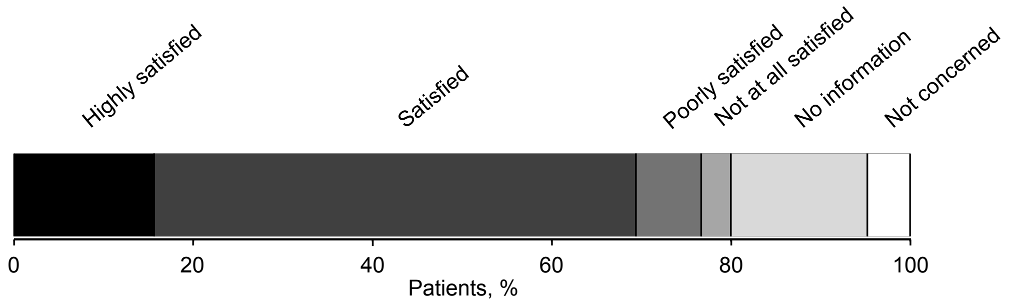

Q9: Was your primary-care physician informed of your hospitalization?

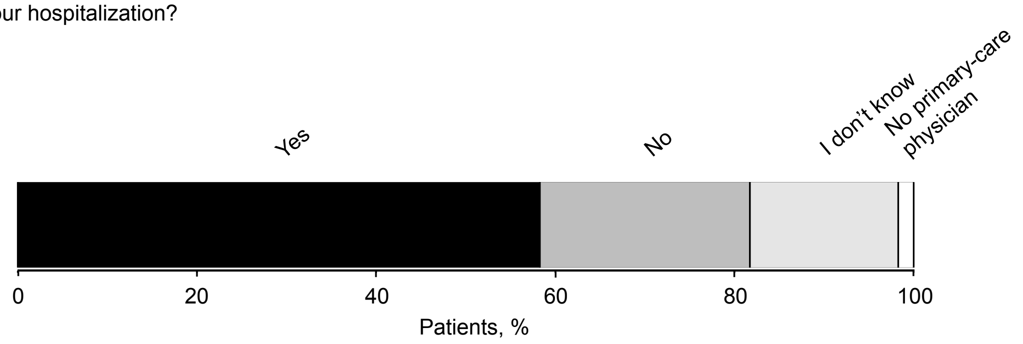

Q10: Did you have the phone number of the unit in which you were hospitalized?

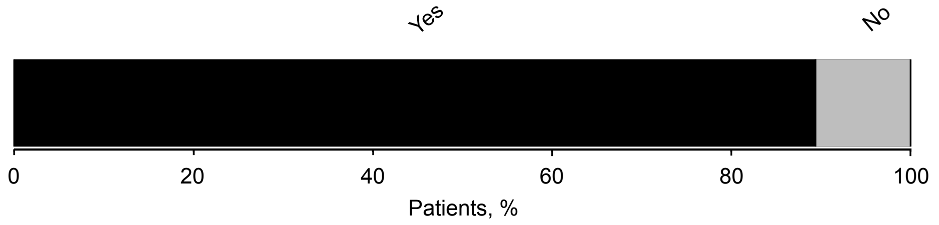

Figure A3-3

Item 3 score: patients' impressions of the hospital-discharge process (Q11A, Q11B, Q11F, Q11G).

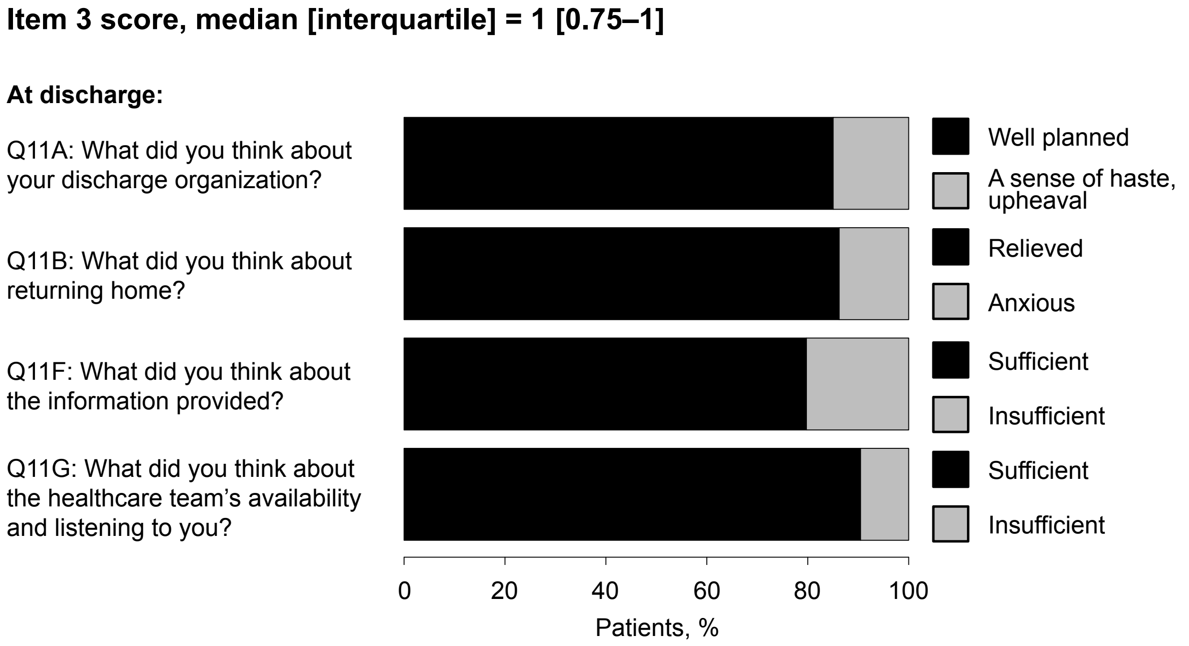

Supplement: Multimedia Appendix 3 [file jmir_v17i6e158_app3.pdf]
